# Using four different clinical tools as predictors for pain after total hip arthroplasty: a prospective cohort study

**DOI:** 10.1186/s12871-020-00959-2

**Published:** 2020-03-03

**Authors:** Anja Geisler, Josephine Zachodnik, Jens Laigaard, Laura S. Kruuse, Charlotte V. Sørensen, Magnus Sandberg, Eva I. Persson, Ole Mathiesen

**Affiliations:** 1 Department of Anesthesiology, Zealand University Hospital, Lykkebækvej 1, 4600 Koege, Denmark; 2 Department of Health Sciences, Faculty of Medicine, Lund University, Lund, Sweden; 3 Department of Orthopaedics, Zealand University Hospital, Koege, Denmark; 4 Department of Clinical Medicine, Faculty of Health Sciences, Copenhagen University, Copenhagen, Denmark

**Keywords:** Postoperative pain, Prediction, Total hip arthroplasty

## Abstract

**Background:**

Treatment of postoperative pain remains a significant clinical problem, and prediction of patients with a risk of higher postoperative pain levels is an important focus. We aimed to identify patients undergoing total hip arthroplasty (THA) with risk of higher pain levels at 24 h postoperatively by using four simple and easily available clinical tools.

**Methods:**

This prospective observational cohort study included 102 patients having THA at Zealand University Hospital in Denmark. The following predictive tools were investigated for identifying patients with higher postoperative pain levels at 24 h postoperatively, both at rest and during mobilization: preoperative pain by peripheral venous cannulation (PVC) (dichotomized according to numerical rating scale pain ≤ 2/> 2 (PVC-Low/PVC-High) (primary outcome); the post anesthesia care unit (PACU) nurses’ expectations of patients pain levels; patients early pain levels at the PACU; and patients own forecast of postoperative pain levels. The Mann-Whitney U test was used to analyze comparisons between prediction groups. For the primary outcome we considered a *p*-value < 0.01 as statistically significant and for other outcomes a p-value of 0.05.

**Results:**

We found no significant differences between the PVC groups for pain during mobilization at 24-h postoperatively: PVC-Low: 6 (4–8) (median, (IQR)) versus PVC-High: 7 (5–8) (median, (IQR)), *p* = 0.10; and for pain at rest: PVC-Low 2 (0–3) (median, (IQR)) versus PVC-High 3 (2–5) (median, (IQR)), *p* = 0.12. Other comparisons performed between predictive groups did not differ significantly.

**Conclusions:**

In this prospective cohort study of 102 THA patients, we did not find that preoperative pain by PVC, when using a cut-off point of NRS ≤ 2, were able to predict postoperative pain at 24 h postoperatively. Neither did PACU nurses’ prediction of pain, patients forecast of pain, nor did maximum pain levels at the PACU.

**Trial registration:**

Retrospectively registered 20th February 2018 at ClinicalTrials.gov (NCT03439566).

## Background

Despite considerable efforts in optimizing postoperative pain, this clinically important symptom remains a major challenge [1]. It is therefore important to identify individuals at risk of developing high postoperative pain levels, but clinically useful predictive tools are virtually absent [2].

A newer study indicated that pain intensity by preoperative peripheral venous cannulation (PVC), using a grouping according to numerical rating scale pain (NRS) ≤ 2 and > 2, was associated with pain levels at the post anesthesia care unit (PACU) [3]. This study, however, did not investigate the prediction of pain later at the surgical ward, which is particularly relevant, since sufficient pain treatment is a cornerstone for optimal rehabilitation [4].

Nurse’s prediction of patient outcomes has been investigated in different settings but with varying results [5, 6]. Therefore, it is relevant to investigate if experienced nurses at the PACU can predict which patients will suffer from higher levels of pain after PACU discharge.

As patients may be predisposed to certain levels of postoperative pain due to e.g. sex, preoperative pain, genetic variations [7], anxiety, or type of surgery [8, 9], it could be relevant to investigate if patient’s pain levels at the PACU, using moderate to severe pain (NRS > 3) as an indicator, can predict pain levels after PACU discharge.

A recent study reported that 47% of patients correctly predicted their pain levels 2 weeks after hand surgery, but they did not investigate the prediction of acute postoperative pain [10]. A subsequent commentary pointed out, that further prospective studies are needed regarding patient’s ability to forecast their disability and pain [11].

Total hip arthroplasty (THA) is a surgical procedure of which patients’ have reported moderate to severe postoperative pain [12]. Therefore, this population is relevant for investigating which patients are high pain responders including a particular focus on pain during recovery at the surgical ward. With this study, we hypothesized that different clinical parameters, nursing staff impressions and patients forecast could be used to predict postoperative pain. This study aimed to investigate if preoperative pain by PVC could be used to identify groups of THA patients with higher levels of pain during mobilization at 24 h postoperatively (primary outcome). Additionally, that PACU nurses’ capability of predicting patients with higher pain levels at the ward, pain levels at the PACU, and patients’ forecast preoperatively, could be used to identify patients with higher postoperative pain levels at 24 h after THA (secondary outcomes).

## Methods

This prospective observational cohort study was approved by the Danish Data Protection Agency (REG-158-2017) and first posted at ClinicalTrials.gov (NCT03439566) on 20th February 2018. The Research Ethics Committee of the Capital Region was consulted, but approval was not needed according to Danish law (Reg. nr. J.nr. 17–000048). Consecutive data was collected at Zealand University Hospital, Koege (ZUHK) in the period from January 2018 to February 2019. The head nurse and chief physician from the orthopaedic department at ZUHK accepted departmental participation in the study, including the collection of patient data. The manuscript follows the STrengthening the Reporting of Observational Studies in Epidemiology (STROBE) statement guidelines [13]. The participants were enrolled after giving verbal and written informed consent when attending the pre-scheduled surgical and anesthetic information meeting about 2 weeks before to surgery.

### Participants

The participants were enrolled after giving verbal and written informed consent when attending the pre-scheduled surgical and anesthetic information meeting about 2 weeks before to surgery.

Inclusion criteria were: age ≥ 18 years old, speaking Danish and/or English, and scheduled for primary elective THA in spinal anesthesia. Exclusion criteria were: not able to-cooperate according to investigators judgement, alcohol or drug abuse, or if correct placement of the PVC was impossible. No investigational intervention was initiated during the study. The department followed the usual local protocols for postoperative pain treatment and standard of care. Two surgical specialists performed all the THA procedures. The lateral surgical approach was used for all patients.

### Outcomes

Primary outcome was: numeric rating scale (NRS) pain (0–10) during mobilization at 24 h postoperatively.

Secondary outcomes were: NRS-pain at rest at 24 h postoperatively, and 24 h intravenous morphine equivalent consumption, mg.

A correctly placed PVC was defined as a cannula placed in a vein on the dorsum part of the dominant hand. The NRS score was performed during the first attempt otherwise the patients were excluded. The allowed cannula sizes were 20G or 22G.

### Anesthesia and analgesic treatment

All patients received spinal anesthesia (10–15 mg bupivacaine). The standard analgesic treatment that was provided for patients at the hospital was: perioperative methylprednisolone IV 125 mg (after induction of anesthesia). At the POTA patients were supplied with opioids as needed, according to usual practice. At the ward, postoperatively, paracetamol 1000 mg OR every 6 h, and slow-release oxycodone 10 mg OR administrated twice a day, supplemented with oxycodone IV as needed.

### Psychological profile

Patients’ psychological profile and relation to pain were tested using the validated self-administered questionnaire, Pain Catastrophizing Scale (PCS). The scale includes 13 items and assesses the extent of the patient’s catastrophizing thoughts and emotions associated with pain. Such thoughts or feelings are rated from zero (not at all) to five (all the time). The PCS has a maximum score of 52. A clinically relevant cut off for being a pain catastrophizer was considered as numbers above 30 [14].

### Supplemental data regarding PACU nurses’prediction

When PACU nurses were asked to state if they predicted a patient to be a high pain responder or not, they were also asked to tick a box with the following statements underpinning their choice: patients’ appearance, patients’ pain intensity, my own intuition, patients’ need of opioids, the patient’s expression of concern and anxiety, or optional additional information – described in their own words.

### Collection of data

For evaluation of pain, the NRS-scale was used, 0 to 10 (0 = no pain and 10 = worst imaginable pain). All patients were instructed preoperatively in how to use the NRS tool [15]. The patients stated their own pain. All data were entered directly into the patient Case Report Form (CRF). All opioids were converted to IV morphine equivalents (eqv) (Additional file 1).

At inclusion, patients completed the PCS questionnaire and provided information about years of education after high school, civil status, employment, as well as their forecast of pain levels. The anesthesia nurse at the operation theater, who performed the peripheral cannulation, asked patients’ about the levels of NRS pain after placement of the PVC. The nurses on the PACU collected data on NRS pain after the spinal anesthesia had ceased, as well as performed a prediction of which patients they believed would experience moderate or severe pain at 24 h during mobilization. The primary investigator, a project nurse or research assistants performed the data collection at the ward at 24 h +/− 2 h postoperatively for pain and opioid consumption.

The following information was registered from the electronic patient records. Preoperative data: height, weight, sex, American Society of Anesthesiologists physical score (ASA), usual preoperative analgesic consumption and pre-medication. Perioperative data: analgesic and antiemetic treatment, duration of surgery. Postoperative data: analgesics used from 0 h to 24 h postoperatively, and length of stay (LOS). All data were registered in the CRF, imported to the statistical package IBM SPSS version 25, and the final data set was double-checked for errors.

The patients also filled out a diary from postoperative day one to five at home regarding pain, side effects, use of analgesics and quality of sleep. These data will be reported elsewhere.

### Sample size and statistics

A sample size estimation was performed for NRS pain during mobilization based on data from a prior study that investigated a similar patient population [12]. To reduce the risk of spurious significant findings, we choose an alfa = 0.01 and a power of 0.9. Furthermore, we used a standard deviation (SD) of 2.5. We found 93 patients were needed to detect a minimal clinically important difference (MCID) of NRS-pain at 1.0. To compensate for the uncertainty of SD we aimed to include 100 consecutive patients undergoing THA.

For comparisons we defined groups based on the following:

1. Pain by preoperative PVC dichotomized to NRS ≤ 2 (PVC-Low) or NRS > 2 (PVC-High)
2. PACU nurses’ prediction of patients being a high pain responder or not (Nurse-Low, or Nurse-High)
3. Maximum NRS pain at the PACU dichotomized to NRS ≤ 3 / > 3 (when spinal anesthesia has ceased, Bromage score 0─1) (PACU-NRS ≤ 3, or PACU-NRS > 3)
4. Patients reporting of being a high pain responder or not (Forecast-Low, or Forecast-High)

Normal distribution was tested visually in histograms and Q-Q plots and quantitatively with Kolmogorov-Smirnov test. Data are presented with either numbers or percentages, median (IQR), mean (SD), and 95% CI, where relevant. Mann-Whitney U test (for non-parametric data) was used to analyze all comparisons between groups: PVC-High versus PVC-Low, Nurse-Low versus Nurse-High, Forecast-Low versus Forecast-High, and PACU-NRS ≤3 versus PACU-NRS > 3. For the primary outcome we considered a *p*-value < 0.01, and for other outcomes, a p-value < 0.05, as statistically significant. Bonferroni correction was used to counteract for mass-significance where relevant.

We performed an exploratory multiple linear regression, both adjusted an unadjusted analyses, using a dependent variable, NRS pain at 24 h during rest or mobilization, and adjusting for the following pre-defined covariates: sex, age, patients pain threshold, marital status, education, daily analgesic consumption, PCS, and employment status. To test for the possibility of multicollinearity, Pearson r for parametric data and Spearman rho for non-parametric data was used.

For evaluating and comparing predictive models we calculated Receiver Operating Characteristic curves (ROC). The true positive rate in the model (sensitivity) was plotted against the false positive rate (1 – specificity) for a given cut-off value of the predictive variable, thus aiming to determine the optimal cut-off value. Areas in the interval 0.9–1 represented excellent prediction, 0.8–0.9 good prediction, 0.7–0.8 fair prediction and 0.6–0.7 poor prediction [16, 17]. Statistical analyzes were expressed using IBM SPSS software version 25 for Windows (SPSS Inc. Chicago, IL).

## Results

**Fig. 1:**
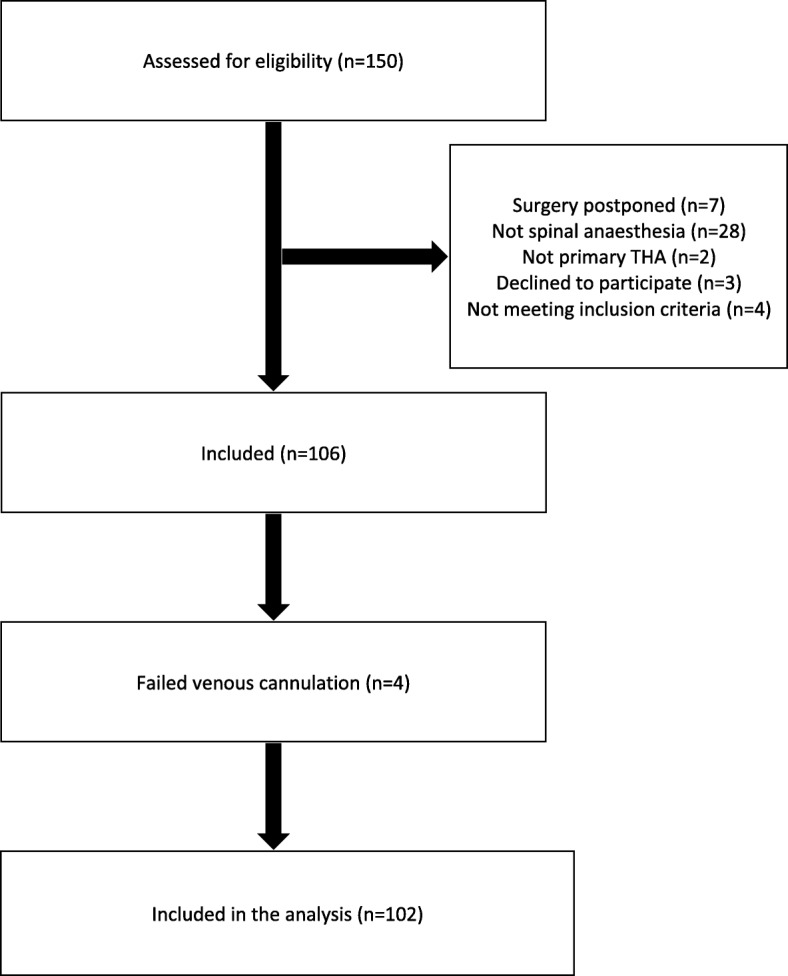
Patient flow

One hundred and fifty patients scheduled for THA were assessed for eligibility. After exclusions, 102 patients were included in the study for evaluation of the primary outcome. For further information see Fig. 1.

The study included 35 males and 67 females, mean age was 69 (19) (mean, (SD)) years, the surgery lasted in 53 (46–63) (median, (IQR)) min, and median LOS was 1 (1, 2) day. For further information, see Table 1.

**Table 1:** Demographics and baseline data

	Total population *n* = 102	Missing (*n*)	PVC-Low (*n* = 67)	Missing (*n*)	PVC-High (*n* = 35)	Missing (*n*)	PVC-Low vs High *p*-value
Sex m/f, (n)	35/67	0	26/41	0	9/26	0	0.18
Age, yr, mean (SD)	69 (19)	0	71 (8)	0	66 (10)	0	0.02
Height, cm, mean (SD)	169 (8)	15	168 (8)	11	169 (8)	4	0.58
Weight, kg, median (IQR)	75 (65–85)	15	75 (64–83)	11	75 (66–98)	4	0.41
ASA 1/2/3 (n)	21/62/16	3	15/40/10	2	6/22/6	1	0.57
Education after high school (no/ yes), (n)	24/71	7	16/46	5	8/25	2	0.98
Civil status (married/^a^living alone) (n)	73/ 29	0	48/19	0	25/10	0	0.06
Employed (no/yes), (n)	72/30	0	51/16	0	21/14	0	0.72
Patients forecast (high pain responder/ normal responder) (n)	21/79	2	16/49	2	5/30	0	0.70
Daily use of any analgesics (no/yes), (n)	47/52	3	28/37	2	19/15	1	0.67
PCS (0–52), median (IQR)	14 (7–21)	0	13 (6–18)	0	17 (12–28)	0	0.91
PCS ≤30 / > 30 (n)	87/15	0	58/9	0	29/6	0	
Surgery time (min), median (IQR)	53 (46–63)	0	53 (47–63)	0	52 (44–64)	0	0.91
Length of stay (days), median (IQR)	1 (1–2)	0	1 (1–2)	0	2 (1–2)	0	0.01

*PVC* Peripheral venous cannulation, *ASA* American Society of Anesthesiologist classification, *PCS* Pain Catastrophizing Scale, ^a^Living alone: Divorced, single, widowed, or not in a relationship

### PVC related pain

For the primary outcome, NRS pain during mobilization at 24 h, we found no significant difference between groups PVC-Low 6 (4–8) (median (IQR)) and PVC-High 7 (5–8) (median (IQR)) (*P* = 0.10) (Table 2). For NRS pain at 24 h at rest, we found no significant difference between groups PVC-Low 2 (0–3) (median (IQR) and PVC-High 3 (2–5) (median (IQR), *p* = 0.12. For total 24 h IV morphine eqv. Consumption, we found no significant difference between groups PVC-Low 20 (15–24) (median (IQR)) mg and Group PVC-High 23 (15–28) (median (IQR) mg (median (IQR), *p* = 0.20 (Table 2).

**Table 2:** All patients and comparisons between predictive groups

	All patients (*n* = 102)	PVC-Low (*n* = 67)	PVC- High (*n* = 35)	p-value	Nurse-Low (*n* = 49)	Nurse-High (*n* = 32)	p-value	PACU-NRS ≤ 3 (*n* = 90)	PACU-NRS > 3 (*n* = 12)	p-value	Forecast-Low (*n* = 79)	Forecast-High (*n* = 21)	*p*-value
Pain (mobilization) 24 h postop.	6 (4–8)	6 (4–8)	7 (5–8)	0.10	5 (4–8)	6 (4–7)	0.78	5 (4–8)	7 (6–8)	0.74	6 (4–8)	6 (4–8)	0.79
Pain (at rest) 24 h postop.	2 (0–4)	2 (0–3)	3 (2–5)	^a^0.12	2 (0–4)	2 (0–4)	0.65	2 (0–4)	3 (2–5)	0.22	2 (1–4)	2 (0–3)	0.19
Morphine consumption (eqv.), IV, mg, (0-24 h)	20 (15–25)	20 (15–24)	23 (15–28)	0.20	19 (15–23)	22 (15–29)	0.16	20 (15–25)	26 (18–33)	^a^0.12	20 (15–28)	20 (15–23)	0.35

^a^Bonferroni correction. *PVC* Peripheral Venous Cannulation. *PACU* Post Anesthesia Care Unit. *NRS* Numerical Rating Scale. Data are median and interquartile range (IQR), pain are numerical rang scale (NRS). Nurse-Low means patients that the PACU nurse evaluates to be an ordinary pain responder and Nurse-High was evaluated to be a high pain responder. Forecast-Low means ordinary pain responder and Forecast-High means high pain responder, according to evaluation by patients themselves before surgery

Explorative regression analyses were performed regarding the association between PVC-Low and PVC-High, and postoperative 24 h NRS pain during rest and mobilization, in both unadjusted and adjusted analyses. For 24 h NRS pain during mobilization, the unadjusted analyses demonstrated no significant difference in standardized Beta 0.88 (− 0.18; 1.94) NRS (95% CI) (*p* = 0.10) between groups defined by PVC pain. When adjusted for sex, age, patient’s forecast of own pain, marital status, education, daily analgesic consumption, PCS, and employment status, we found a significant difference in standardized Beta NRS (95% CI) 0.24 (0.14; 2.43) (*p* = 0.03) between the groups defined by pain during PVC (Table 3). For NRS pain at rest at 24 h the unadjusted Beta showed a significant difference in NRS (95% CI), 1.13 (0.14; 2.12) (p = 0.03) between groups defined by PVC pain. In the adjusted analyses, however, this difference became non-significant with a standardized Beta NRS (95% CI) 0.18 (− 0.22; 2.06) (*p* = 0.11) (Table 3). We did not find any multicollinearity of parameters in the adjusted analyses.

**Table 3:** Multiple linear regression model for NRS pain by PVC. Adjusted and unadjusted

Dependent variable	Independent variable	Beta Estimate NRS (95% CI)	*P*-value	R^2^
Pain at 24 h (mobilization) Unadjusted	NRS by PVC ≤/> 2	0.88 (− 0.18;1.94)	0.10	0.27
Pain at 24 h (mobilization) Adjusted	NRS by PVC ≤/> 2	0.24^a^(0.14;2.43)	0.03	0.20
Pain at 24 h (at rest) Unadjusted	NRS by PVC ≤/> 2	1.13 (0.14;2.12)	0.03	0.05
Pain at 24 h (at rest) Adjusted	NRS by PVC ≤/> 2	0.18^a^(−0.22;2.06)	0.11	0.13

Adjusted for; sex, age, patients pain threshold, marital status, education, daily analgesic consumption, PCS and employment

*NRS* Numerical Rating Scale. *PVC* Peripheral Venous Cannulation. *CI* Confidence Interval ^a^Standardized Beta value

### PACU nurse prediction

We found no significant differences between groups Nurse-Low and Nurse-High for NRS pain during mobilization at 24 h postoperatively Nurse-Low: 5 (4–8) and Nurse-high: 6 (4–7) (median (IQR)), *p* = 0.78. No significant differences between groups were found for pain at rest at 24 h postoperatively, and for 24 h morphine consumption. See Table 2 for further details.

### Pain at the PACU

For groups based on NRS pain ≤ 3 and NRS pain > 3 at the PACU, we found no significant differences between groups, for pain during mobilization at 24 h postoperatively, PACU-NRS ≤ 3: 5 (4–8) and PACU-NRS > 3: 7 (6–8) (median (IQR)), *p* = 0.74. No significant differences between groups were found for pain at rest. For 24 h morphine consumption, we found a significant difference between group PACU-NRS ≤ 3: 20 (15–25) mg and PACU-NRS > 3: 26 (18–33) mg (median (IQR)), *p* = 0.03, Bonferroni adj. See Table 2 for details.

### Patients forecast of pain

We found no significant differences between groups Forecast-Low and Forecast-High regarding NRS pain during mobilization at 24 h postoperatively; Forecast-Low: 6 (4–8) and Forecast-High 6 (4–8) (median (IQR)), *p* = 0.79. No significant differences between groups for NRS pain 24 h during rest and opioid consumption was detected. For further information see Table 2.

### ROC curve analyses

We performed ROC curves for groups PVC-Low and PVC-High, and its capability of predicting pain during rest and mobilization 24 h, as well as 24 h opioid consumption. Since all ROC had AUC values less than 0.60, we did not consider these to be reliable predictors (Additional file 2).

## Discussion

With this prospective observational cohort study of 102 patients undergoing THA, we investigated four simple and easily available clinical tools to predict patients with higher levels of postoperative pain 24 h after surgery. We did, however, not find any significant difference for postoperative pain during mobilization, at rest, between groups of patients defined by pain by PVC, PACU nurses’ prediction, pain levels at the PACU, and patients forecast of pain.

Pain from preoperative peripheral venous cannulation was previously investigated (3) for prediction of postoperative pain in a study with 180 patients undergoing laparoscopic cholecystectomy. Here, Persson and colleagues (3) reported that patients with NRS pain > 2 by PVC reported higher pain scores at rest and received more opioids within the first 90 min at the PACU, compared to those with NRS ≤ 2. Furthermore, in a newer and larger study, Persson and co-workers [18] repeated these findings, but this time in a population of 600 patients undergoing different surgical procedures, receiving different types of anesthesia, and using different places for venous cannulation. Still, they reported that NRS pain > 2 during PVC was associated with moderate to severe postoperative pain at rest at the PACU. We could not confirm these findings, as we found no significant differences for pain at rest at the PACU between PVC-Low and PVC-High. Our study differed from those of Persson and colleagues’ in several ways, including different patient populations, differences in age, and the type of anesthesia. Also, we measured pain both at rest and during mobilization, and not only at the PACU but also 24 h postoperatively. We did not find any association between PACU nurses’ prediction and patients’ pain levels after 24 h, neither at rest nor during mobilization. Interestingly, in the follow-up on background for their choice, we found that the PACU nurses stated that the forecasts of patients being low or a high pain responder, to be primarily based upon the appearance of the patient and on their intuition. These findings were supported by other studies demonstrating that when nurses assess if patients are in pain or not, they primarily base their decision on patients’ appearances and their non-verbal behavior [19, 20]. However, this failed to show applicability in this study.

In a recent study in 563 women having breast cancer surgery, Sipilä and colleagues [21] found that patient’s expectations of severe postoperative pain were associated with higher clinical pain intensity and increased initial oxycodone use at the PACU. In contrast, in another and smaller prospective study [10] investigating patients’ ability to forecast their disability and pain two weeks after hand surgery, only weak correlations between forecasted and realized pain was discovered [10]. In contrast, we did not find any differences in patients pain scores nor opioid usage for the first 24 h postoperatively, based on patients’ forecast on being a high pain responder or not. It is possible that differences in type of surgeries and patient populations between these and the present study influences the differences in outcomes, and further studies are needed to elaborate on this clinically relevant topic.

### Strengths and limitations

In this prospective study it was a strength that all patients had spinal anesthesia and underwent the same surgical procedure, which was performed by the same two orthopaedic surgeons, and with all patients receiving a standardized perioperative pain treatment. This minimizes variations and bias. Furthermore, PVC was performed by experienced anesthesia nurses, and all were placed in the patients’ dominant hand. Also, data at the ward were collected by a limited group of four investigators, reducing observer bias.

We used a cut-off point of NRS 2, for the division of groups, based on the study by Persson et al. [3]. The ROC curve analyses indicated that a cut-off point of NRS 2.5 could have been more appropriate for dividing patients into groups (Additional file 2). However, the AUC-value of the ROC was low and of limited reliability. Missing data was a limitation, especially the relatively large proportion of missing data on PACU nurses’ prediction could have influenced our results. Preoperative pain may serve as a predictor for postoperative pain levels [8]. It could therefore be considered as a limitation, that we did not register patients’ preoperative pain, but instead, as a proxy here for, registered preoperative analgesic consumption. Another limitation is the sample size calculation, as such calculations typically are based on an equal number of patients in the investigated groups. This was unfortunately not the case, as number of patients differed between the four investigated groups of predictors. As this was a clinical prospective cohort, and not a randomized trial, it can be argued that sample sizes are of limited value, especially when we did not have influence on the distribution of number of patients in the compared groups. Our study can, however, serve as base for sample size calculations of future studies, preferably including larger numbers of participants.

In perspective, the prediction of postoperative pain levels continues to be an important focus for future research, as individualized pain treatment has the potential to improve patient courses. Such research may well be based on the collection of big data including new types of analyses hereof, biomarkers, neuroimaging, physiological and psychological variables, and clinical data as well. It is possible, that results of studies using simple clinical tools, in the future, might be included in such big data in the guidance of pain treatment. Until then, the focus must be on effective pain treatment, regular assessment of pain with frequently monitoring of patients and their needs.

## Conclusions

In this prospective cohort study of 102 THA patients, we did not find that preoperative pain by PVC, when using a cut-off point of NRS ≤ 2, were able to predict postoperative pain at 24 h after THA postoperatively. Neither did PACU nurses’ prediction of pain, patients forecast of pain, nor did maximum pain levels at the PACU.

## Supplementary information

**Additional file 1.** Supplemental Digital Content 1: Opioid conversion.

**Additional file 2.**

## Data Availability

The datasets used and/or analyzed during the current study are available from the corresponding author on reasonable request.

## Abbreviations

ASA: American Society of Anesthesiologists physical score
CRF: Case Report Form
Eqv: Equivalents
IQR: Inter Quartile Range
IV: Intravenous
LOS: Length of stay
NRS: Numeric Rating Scale
PACU: Post Anesthesia Care Unit
PCS: Pain Catastrophizing Scale
PVC: Peripheral Venous Cannulation
RCT: Randomized controlled trial
THA: Total Hip Arthroplasty
VAS: Visual Analogue Scale
VRS: Verbal Rating Scale
ZUHK: Zealand University Hospital, Koege

## References

[1] Gerbershagen HJ, Aduckathil S, van Wijck AJM, Peelen LM, Kalkman CJ, Meissner W (2013). Pain intensity on the first day after surgery: a prospective cohort study comparing 179 surgical procedures. Anesthesiology..

[2] Werner MU, Mjöbo HN, Nielsen PR, Rudin A (2010). Prediction of postoperative pain: a systematic review of predictive experimental pain studies. Anesthesiology..

[3] Persson AK, Pettersson FD, Dyrehag L-E, Åkeson J (2016). Prediction of postoperative pain from assessment of pain induced by venous cannulation and propofol infusion. Acta Anaesthesiol Scand.

[4] Kehlet H, Dahl JB (2003). Anaesthesia, surgery, and challenges in postoperative recovery. Lancet..

[5] Lipson Amy R, Miano Sarah J, Daly Barbara J, Douglas SL (2017). The Accuracy of Nurses’ Predictions for Clinical Outcomes in the Chronically Critically IllNo Title. Res Rev J Nurs Heal Sci.

[6] Zachariasse JM, Van Der Lee D, Seiger N, De Vos-Kerkhof E, Oostenbrink R, Moll HA (2017). The role of nurses’ clinical impression in the first assessment of children at the emergency department. Arch Dis Child.

[7] Horjales-Araujo E, Dahl JB. Is the experience of thermal pain genetics dependent? Biomed Res Int. 2015;2015:349584. 10.1155/2015/349584 PMC4324494 25699274

[8] Yun H, Ip V, Abrishami A, Peng PWH, Wong J, Chung F (2009). Predictors of postoperative pain and analgesic consumption a qualitative systematic review. Anesthesiology..

[9] Aubrun F, Salvi N, Coriat P, Riou B (2005). Sex- and age-related differences in morphine requirements for postoperative pain relief. Anesthesiology.

[10] Alokozai A, Eppler SL, Lu LY, Sheikholeslami N, Kamal RN (2019). Can patients forecast their postoperative disability and pain?. Clin Orthop Relat Res.

[11] Vranceanu A-M (2019). CORR insights®. Clin Orthop Relat Res.

[12] Geisler A, Dahl JB, Thybo KH, Pedersen TH, Jørgensen ML, Hansen D, et al. Pain management after total hip arthroplasty at five different Danish hospitals: A prospective, observational cohort study of 501 patients. Acta Anaesthesiol Scand. 2019; Available from: https://onlinelibrary.wiley.com/doi/abs/10.1111/aas.13349. 10.1111/aas.13349 30883668

[13] von Elm E, Altman DG, Egger M, Pocock SJ, Gøtzsche PC, Vandenbroucke JP (2014). The Strengthening the reporting of observational studies in epidemiology (STROBE) statement: guidelines for reporting observational studies. Int J Surg.

[14] Sullivan M, Bishop S, Pivik J (1995). The pain catastrophizing scale: user manual. Psychol Assess.

[15] Hjermstad MJ, Fayers PM, Haugen DF, Caraceni A, Hanks GW, Loge JH (2011). Studies comparing numerical rating scales, verbal rating scales, and visual analogue scales for assessment of pain intensity in adults: a systematic literature review. J Pain Symptom Manage.

[16] Landis JR, Koch GG (1977). Landis_Jr__Koch_Gg_1977_Kappa_and_Observer_Agreement. Biometrics..

[17] Pepe MS, Janes H, Longton G, Leisenring W, Newcomb P (2004). Limitations of the odds ratio in gauging the performance of a diagnostic, prognostic, or screening marker. Am J Epidemiol.

[18] Persson AKM, Åkeson J (2019). Prediction of acute postoperative pain from assessment of pain associated with venous Cannulation. Pain Pract.

[19] Drayer RA, Henderson J, Reidenberg M (1999). Barriers to better pain control in hospitalized patients. J Pain Symptom Manag.

[20] Schafheutle EI, Cantrill JA, Noyce PR (2001). Why is pain management suboptimal on surgical wards?. J Adv Nurs.

[21] Sipilä RM, Haasio L, Meretoja TJ, Ripatti S, Estlander AM, Kalso EA (2017). Does expecting more pain make it more intense? Factors associated with the first week pain trajectories after breast cancer surgery. Pain..

