# Supplementary material for: Using four different clinical tools as predictors for pain after total hip arthroplasty: a prospective cohort study
Source: BMC Anesthesiol. 2020 Mar 3;20:57. doi: 10.1186/s12871-020-00959-2 (PMC7055106; doi:10.1186/s12871-020-00959-2)
Supplement: Supplementary file 1 — Additional file 1. Supplemental Digital Content 1: Opioid conversion. [file 12871_2020_959_MOESM1_ESM.docx]

Appendix 1:

# Supplemental Digital Content 1: Opioid conversion

| **Opioid** | **Administration** | **Opioid: Intravenous morphine** |
| --- | --- | --- |
| 1 mg Fentanyl | i.v. | 100 mg morphine |
| 1 mg Morphine | oral | 0.33 mg morphine |
| 1 mg Oxycodone | i.v. | 1.33 mg morphine |
| 1 mg Oxycodone | p.o | 0.5 mg morphine |
| 1 mg Sufentanil | i.v. | 1000 mg morphine |
| 1 mg Tramadol | oral | 0.07 mg morphine |
