# Supplementary material for: Using four different clinical tools as predictors for pain after total hip arthroplasty: a prospective cohort study
Source: BMC Anesthesiol. 2020 Mar 3;20:57. doi: 10.1186/s12871-020-00959-2 (PMC7055106; doi:10.1186/s12871-020-00959-2)
Supplement: Supplementary file 2 — Additional file 2. [file 12871_2020_959_MOESM2_ESM.docx]

Additional file 2

Area under the curve: 0.40


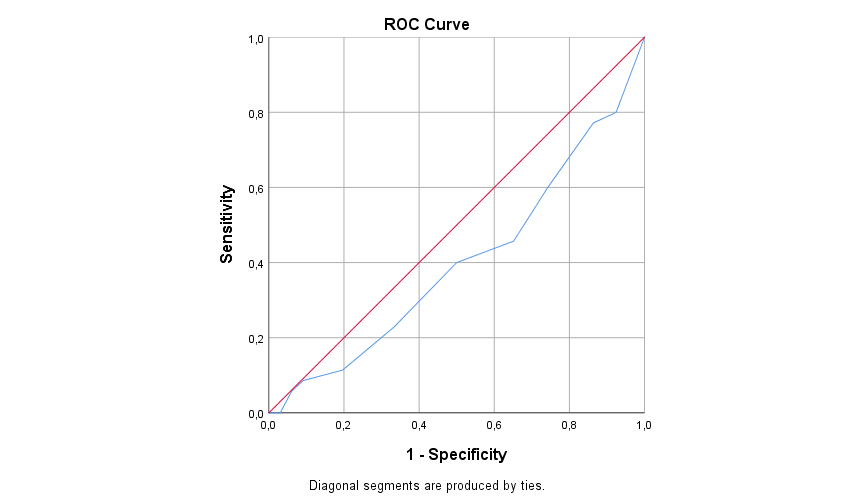


*Receiver-operating characteristics curve for venous cannulation (PVC) as a predictor for 24hr pain during mobilisation*

Area under the curve 0.37


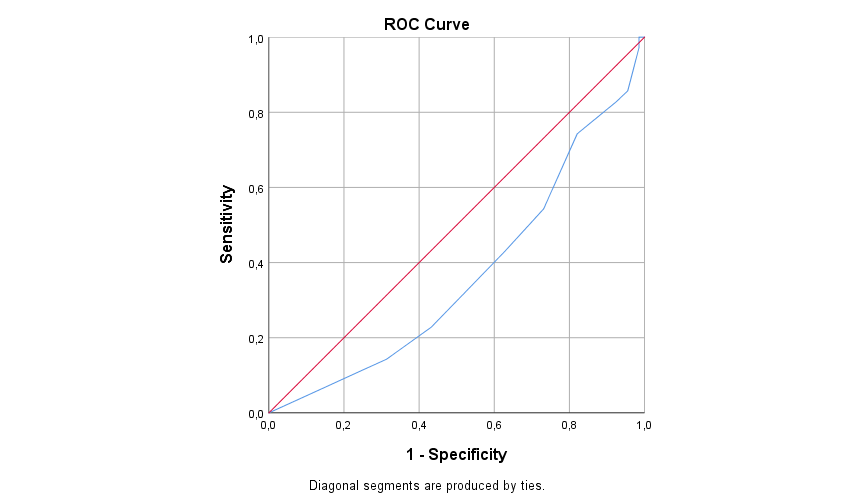


*Receiver-operating characteristics curve for venous cannulation (PVC) as a predictor for 24hr pain at rest*

Area under the curve 0.42


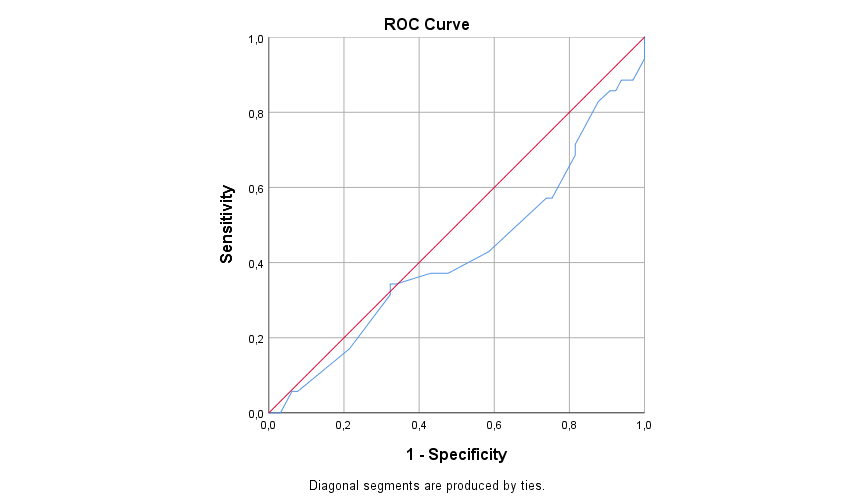


*Receiver-operating characteristics curve for venous cannulation (PVC) as a predictor for 24hr opioid consumption*
